# Characterization of *bla*_AFM-1_-positive carbapenem-resistant strains isolated in Guangzhou, China

**DOI:** 10.1186/s12941-023-00592-0

**Published:** 2023-05-17

**Authors:** Yingcheng Qin, Yuan Peng, Xiaonv Duan, Zhenli Song, Rong Huang, Yongyu Rui

**Affiliations:** 1 Laboratory Medicine Center, Nanfang Hospital, Southern Medical University, Tonghe, Guangzhou, 510515 China; 2 The Second Affiliated Hospital of Guilin Medical University, Guilin, 541199 Guangxi Zhuang Autonomous Region China; 3 Jiangxi Maternal and Child Health Care Hospital of Nanchang Medical College, Nanchang, 330038 Jiangxi Province China

**Keywords:** Class B carbapenemase, AFM-1, Plasmid, Carbapenem-resistant, Mobile genetic elements, Mobilization

## Abstract

**Background:**

Carbapenemase-producing makes a great contribution to carbapenem resistance in Gram-negative bacilli. *Bla*_AFM-1_ gene was first discovered by us in *Alcaligenes faecalis* AN70 strain isolated in Guangzhou of China and, was submitted to NCBI on 16 November 2018.

**Methods:**

Antimicrobial susceptibility testing was performed by broth microdilution assay using BD Phoenix 100. The phylogenetic tree of AFM and other B1 metallo-β-lactamases was visualized by MEGA7.0. Whole-genome sequencing technology was used to sequence carbapenem-resistant strains including the *bla*_AFM-1_ gene. Cloning and expressing of *bla*_AFM-1_ were designed to verify the function of AFM-1 to hydrolyze carbapenems and common β-lactamase substrates. Carba NP and Etest experiments were conducted to evaluate the activity of carbapenemase. Homology modeling was applied to predict the spatial structure of AFM-1. A conjugation assay was performed to test the ability of horizontal transfer of AFM-1 enzyme. The genetic context of *bla*_AFM-1_ was performed by Blast alignment.

**Results:**

*Alcaligenes faecalis* strain AN70, *Comamonas testosteroni* strain NFYY023, *Bordetella trematum* strain E202, and *Stenotrophomonas maltophilia* strain NCTC10498 were identified as carrying the *bla*_AFM-1_ gene. All of these four strains were carbapenem-resistant strains. Phylogenetic analysis revealed that AFM-1 shares little nucleotide and amino acid identity with other class B carbapenemases (the highest identity (86%) with NDM-1 at the amino acid sequence level). The spatial structure of the AFM-1 enzyme was predicted to be αβ/βα sandwich structure, with two zinc atoms at its active site structure. Cloning and expressing of *bla*_AFM-1_ verified AFM-1 could hydrolyze carbapenems and common β-lactamase substrates. Carba NP test presented that the AFM-1 enzyme possesses carbapenemase activity. The successful transfer of pAN70-1(plasmid of AN70) to *E.coli* J53 suggested that the *bla*_AFM-1_ gene could be disseminated by the plasmid. The genetic context of *bla*_AFM_ indicated that the downstream of the *bla*_AFM_ gene was always adjacent to *trpF* and *ble*_MBL_. Comparative genome analysis revealed that *bla*_AFM_ appeared to have been mobilized by an ISCR27-related mediated event.

**Conclusions:**

The *bla*_AFM-1_ gene is derived from chromosome and plasmid, and the *bla*_AFM-1_ gene derived from the pAN70-1 plasmid can transfer carbapenem resistance to susceptible strains through horizontal transfer. Several *bla*_AFM-1_**-**positive species have been isolated from feces in Guangzhou, China.

**Supplementary Information:**

The online version contains supplementary material available at 10.1186/s12941-023-00592-0.

## Background

Carbapenemase-producing organisms hydrolyze carbapenems and most β-lactam antibiotics and pose challenges to clinical diagnostics and therapy. Class B carbapenemases (including subclasses B1, B2, and B3) are metallo-β-lactamases (MBLs) that hydrolyze almost all β-lactam antibiotics requiring Zn^2+^ [1, 2]. However, their activities can suppress by EDTA but cannot inhibit by β-lactamase inhibitors, such as clavulanic acid [2]. B1 subclass carbapenemases have a broader spectrum of drug resistance when compared to B2, are more common than B3 subclass carbapenemases [1], and include clinically significant IMP (Imipenemase metallo-β-lactamase) and VIM (Verona integron-encoded metallo-β-lactamase) metalloenzymes in plasmids, integrons or transposons [1]. In particular, the emergence of NDM (New Delhi metallo-β-lactamase) has caused widespread concern, owing to its high transmission speed and superior drug resistance [3, 4].

Mobile genetic elements (MGEs) often facilitate the spread of carbapenemases, such as plasmids, insertion sequences (IS), integrons and transposons, among others [5, 6]. Several B1 carbapenemases reside on gene cassettes, including *bla*_IMP_, *bla*_VIM_, *bla*_GIM_ among others [6], Many MGEs are responsible for the spread of *bla*_NDM_, including ISAba125, IS26, ISCR1, Tn3, among others [7]. With the help of integrase, integrons can recognize and capture drug-resistance gene cassettes and spread antimicrobial resistance genes (ARGs) through mobile plasmids and transposons [8]. Our previous study reported that several carbapenemase genes can be associated with ISCR1-related variable regions to composite a complex class of integrons [9]. Conjugative plasmids spread B1 carbapenemase genes horizontally to intra- or inter-species bacteria by conjugation assay, which confers resistance to carbapenems.

*Bla*_AFM-1_ (AFM standing for *Alcaligenes faecalis metallo*), was first found in *Alcaligenes faecalis* strain AN70 (*A. faecalis* AN70), and later several Gram-negative strains such as *Comamonas testosteroni* NFYY023 (*C. testosteroni* NFYY023), *Bordetella trematum* E202 (*B. trematum* E202), and *Stenotrophomonas maltophilia* NCTC10498 (*S. maltophilia* NCTC10498) that carried *bla*_AFM-1_ gene were screened out by our LAMP assay [10]. Next, other researchers found that the *bla*_AFM-1_ gene was also identified on the plasmids of an *Aeromonas hydrophila* (*A. hydrophila*) isolate [11] and *Pseudomonas aeruginosa* (*P. aeruginosa*) isolates [12]. Then, novel variants *bla*_AFM-2_, *bla*_AFM-3_, and *bla*_AFM-4_, which differed from *bla*_AFM-1_ due to the C base mutated to T base (C → T) at the position of 44, 37, and 40 at the nucleotide sequence, respectively, which were detected in several carbapenem-resistant *P. aeruginosa* by Yunsong Yu et al. [13, 14].

In this study, we reported a separate B1 carbapenemase, designated to NCBI on 16 November 2018 to get an accession number, we named it AFM-1, which is an acronym for ‘*Alcaligenes faecalis* metallo enzyme’ and exhibited 86% amino acid identity to NDM-1. We screened four species carrying the *bla*_AFM-1_ gene by the LAMP detection method, as previously reported [10]. Intriguingly, some researchers have also found this gene carried by other species [11, 12]. Here, we aim to describe the structure and function of the *bla*_AFM-1_ gene by the following aspects: phylogenetic analysis, amino acid structure, genetic context, horizontal transfer, and cloning and expression experiment, which provide a theoretical basis for the study of the transmission mechanism of the *bla*_AFM-1_ gene.

## Materials and methods

### Strains and antimicrobial susceptibility testing

All strains were recovered from the feces of the inpatient patients. AN70, NFYY023, E202, and NCTC10498 strains were verified as carrying *bla*_AFM-1_ gene in the previous study [10]. AN70, NFYY023, E202, and NCTC10498 strains were grown on plates containing 4 μg/ml meropenem and incubated at 35 °C for 18 h. Antimicrobial susceptibility testing (AST) of isolates was performed by broth microdilution assay and the minimal inhibitory concentration (MIC) values were interpreted by Clinical And Laboratory Standard Institute (CLSI) document M100-32ed [15].

### Phylogenetic analysis and amino acid sequences alignment

All nucleotide acid sequences of known *bla*_AFM_ genes and the common B1 carbapenemases gene (*bla*_NDM-1_(KP772192.1), *bla*_NDM-2_(KU510391.1), *bla*_NDM-3_(JQ734687.1), *bla*_NDM-4_(MG833403.1), *bla*_NDM-5_(KP772210.1), *bla*_NDM-6_(NG049338.1), *bla*_NDM-7_(JX262694.1), *bla*_NDM-8_(AB744718.1), *bla*_NDM-9_(CP021177.1), *bla*_NDM-10_(KF361506.1), *bla*_NDM-11_(KP265940.1), *bla*_NDM-12_(AB926431.1), *bla*_NDM-13_(LC012596.1), *bla*_NDM-14_(NG_049331.1), *bla*_NDM-15_(NG_049332.1), *bla*_NDM-16_(KP862821.1), *bla*_NDM-17_(NG_052662.1), *bla*_NDM-18_(KY503030.1), *bla*_NDM-19_(MF370080.1), *bla*_NDM-20_(KY654092.1), *bla*_NDM-21_(MG183694.1), *bla*_NDM-22_(MH243357.1), *bla*_NDM-23_(MH450214.1), *bla*_NDM-24_(NG_060571.1), *bla*_NDM-25_(NG_066711.1), *bla*_NDM-26_(MK079575.1), *bla*_NDM-27_(MK105832.1), *bla*_NDM-28_(MK425035.1), *bla*_NDM-29_(MF379694.1), *bla*_NDM-30_(MW306748.1), *bla*_NDM-31_(MW306749.1), *bla*_NDM-33_(MZ004933.1), *bla*_NDM-34_(MZ254705.1), *bla*_NDM-35_(MZ265788.1), *bla*_NDM-38_(MZ359766.1), *bla*_NDM-39_(MZ748325.1), *bla*_NDM-40_(MZ748326.1), *bla*_NDM-41_(MZ913436.1), *bla*_NDM-42_(ON205946.1), *bla*_IMP-1_(GQ864268.1), *bla*_SPM-1_(GU831565.1), *bla*_VIM-1_(KP975077.1), *bla*_GIM-1_(NG_049143.1), *bla*_FIM-1_(JX570731.1)) were obtained from the NCBI database and aligned with CLUSTALW. Phylogenetic trees were generated based on the neighbor-joining method using MEGA7.0. The alignment of the secondary structure among subtypes of AFM and NDM-1/6 through visualizer software ESPript 3.0 (https://espript.ibcp.fr/ESPript/cgi-bin/ESPript.cgi).

### Spatial structure prediction of AFM

The spatial structure of the AFM enzyme was generated by homology modeling using the online SWISS-MODEL tool (https://swissmodel.expasy.org/interactive). NDM-1/6 served as a reference enzyme model.

### Whole genome sequencing (WGS)

Total DNA was extracted based on the protocol of Gentra Puregene Yeast/Bact. Kit (QIAGEN, Hilden, Germany) using PacBio (Pacific Biosciences, Menlo Park, CA, USA) and Illumina HiSeq (Illumina, Inc., San Diego, CA, USA) platforms at the Health Time Genomics Institute (Shenzhen, China). Sequencing reads with insert sizes of 20,000 bp and 350 bp were obtained, respectively. The long reads were assembled by de novo assembler (HGAP-v3) to obtain the genome framework sequence [16]. The short paired-end reads were also used to assemble genomes by de novo assembler (SOAPdenovo 2.04) [17], mainly for genome sequence frame correction and single base site correction at the Health Time Gene Institute.

To ensure the accuracy and reliability of the subsequent analysis, sequence correction was performed on the original sequencing data. The genome sequence correction process goes through two steps: (1) use BWA software (http://bio-bwa.sourceforge.net/) to compare the short paired-end reads to the assembled sequence, and then use SAMtools [18] software to identify the different bases on the assembled sequence and correct the wrong bases; (2) After the correction in step (1), the BWA software is used again to compare the reads to the new sequence, and then the Tablet comparison result visualization software [19] is used to display the comparison results. After manual inspection, the accuracy of each site of the assembled sequence is improved, and the wrong sites found again are corrected to obtain the most accurate and no-problem genome sequence. The alignment results of reads and sequence fragments with genomic sequence show that the sequence is complete. Gene predicted by using both GeneMark (version 4.6b, http://topaz.gatech.edu/GeneMark/) and BLAST (https://blast.ncbi.nlm.nih.gov/).

### Cloning and expressing of *bla*_AFM-1_

Using pET-28a( +) plasmid and *E. coli* Top10 competent cells as plasmid vector and expression vector to generate AFM-1 clones (pET-28a( +)-AFM-1-Top10). The specific operation is the same as the previously described [10]. Positive clones were screened with 4 μg/ml meropenem. Sequences analysis confirmed the correct target sequences of positive clones.

### Phenotype of carbapenemase

To assess the activity of carbapenemase, the Carba NP test was carried out by measuring the imipenem hydrolysis in a crude extract of bacteria protein in vitro. Experiment details referred to the previously reported [20]. Using an *Escherichia coli* strain 3 that only harbored *bla*_NDM-1_ carbapenemase gene as the positive control. Two isolates recovered from clinical (*Klebsiella pneumoniae* 10003730 and *Escherichia coli* strain 10004114) which were completely sensitive to common antibiotics (Additional file 1: Tables S1, S2) acted as negative controls. Carba NP data interpretation refers to CLSI guidelines.

The detection of MBL production was performed using an Etest strip according to the manufacturer’s protocol (AB, BIOMERIEUX, Solna, Sweden). The E-test MBL strips were composed of imipenem (4 to 256 µg/ml) and imipenem (1 to 64 µg/ml)-EDTA. The Detailed experiment can be seen in the previous literature [21]. *E.coli* J53 was used as a negative control.

### Conjugation assays

Conjugation assays were carried out in broth using the sodium azide-resistant *E. coli* J53 strain as the recipient. Transconjugants were selected on Mueller–Hinton (MH) agar plates containing 4 μg/ml meropenem and 150 μg/ml sodium azide. To confirm transconjugants, PCR analysis and sequencing were carried out using primers AFM-1-F CGATTGGTGAGCAGGTGGATAAGG and AFM-1-R TCGACAAGGCATTGGCGTAAGTG for *bla*_AFM-1_ PCR screening. Transfer frequency is the number of transconjugants divided by the number of recipient bacteria [22]. The MICs of common beta-lactam antibiotics against the donor, recipient, and transconjugants were checked using E-test strips (BioMérieux SA, La Balme-Les-Grottes, France).

### Comparative genome analysis

ARGs are predicted by the Comprehensive Antibiotic Resistance Database (CARD) and ResFinder database (https://cge.cbs.dtu.dk/services/ ResFinder/), the Mobile genetic element was recognized by ISfinder (https://www-is.biotoul.fr). Plasmid types were identified using the Center for Genomic Epidemiology (http://genomicepidemiology.org/) and BLAST tools. All sequences to be analyzed were downloaded from the NCBI database, and comparative analysis was performed by Blast alignment.

## Results

### AST of target strains

AN70, NFYY023, E202, and NCTC10498 strains were identified by BD Mérieux, and the strains were *A. faecalis, C. testosteroni, B. trematum*,* S. maltophilia,* respectively. AST showed that these strains were multi-drug resistant bacteria. All of these strains are resistant to carbapenems. Detailed AST results of strains are presented in Table 1.

**Table 1:** Antibiotics susceptibility testing of target strains

Antibiotics	AN70 MIC (µg/ml)		NFYY023 MIC		E202 MIC		NCTC10498 MIC	
Amikacin	> 32	R	> 32	R	< = 8	S	16	R
Gentamicin	> 8	R	> 8	R	> 8	R	8	R
Imipenem	> 8	R	> 8	R	> 8	R	> 8	R
Meropenem	> 8	R	> 8	R	> 8	R	> 8	R
Cefazolin	> 16	–	> 16	–	> 16	–	> 16	R
Ceftazidime	> 16	R	> 16	R	> 16	R	> 16	R
Cefotaxime	> 32	R	> 32	R	> 32	R	> 32	R
Cefepime	> 16	R	> 16	R	> 16	R	> 16	–
Ampicillin	> 16	–	> 16	–	> 16	–	> 16	R
Piperacillin	> 64	R	64	I	64	I	> 64	R
Amoxicillin-Clavulanate	> 16/8	R	8/4	R	> 16/8	R	> 16/8	R
Ampicillin-Sulbactam	> 16/8	R	> 16/8	–	> 16/8	–	> 16/8	R
Piperacillin-Tazobactam	> 64/4	R	64/4	I	64/4	I	> 64/4	R
Trimethoprim-Sulfamethoxazole	> 2/38	R	> 2/38	R	> 2/38	R	2/38	S
Chloramphenicol	> 16	R	> 16	R	> 16	R	16	I
Ciprofloxacin	> 2	R	> 2	R	> 2	–	> 2	–
Levofloxacin	> 8	R	> 8	R	> 8	R	> 8	R
Moxifloxacin	> 4	–	> 4	–	> 4	–	> 4	–
Tetracycline	> 8	R	> 8	R	> 8	R	> 8	R

*MIC* minimum inhibitory concentration, *R* resistant, *I* intermediate, *S* susceptible, - No susceptibility breakpoint

### Phylogenetic relationship and secondary structure analysis of AFM

**Fig. 1:**
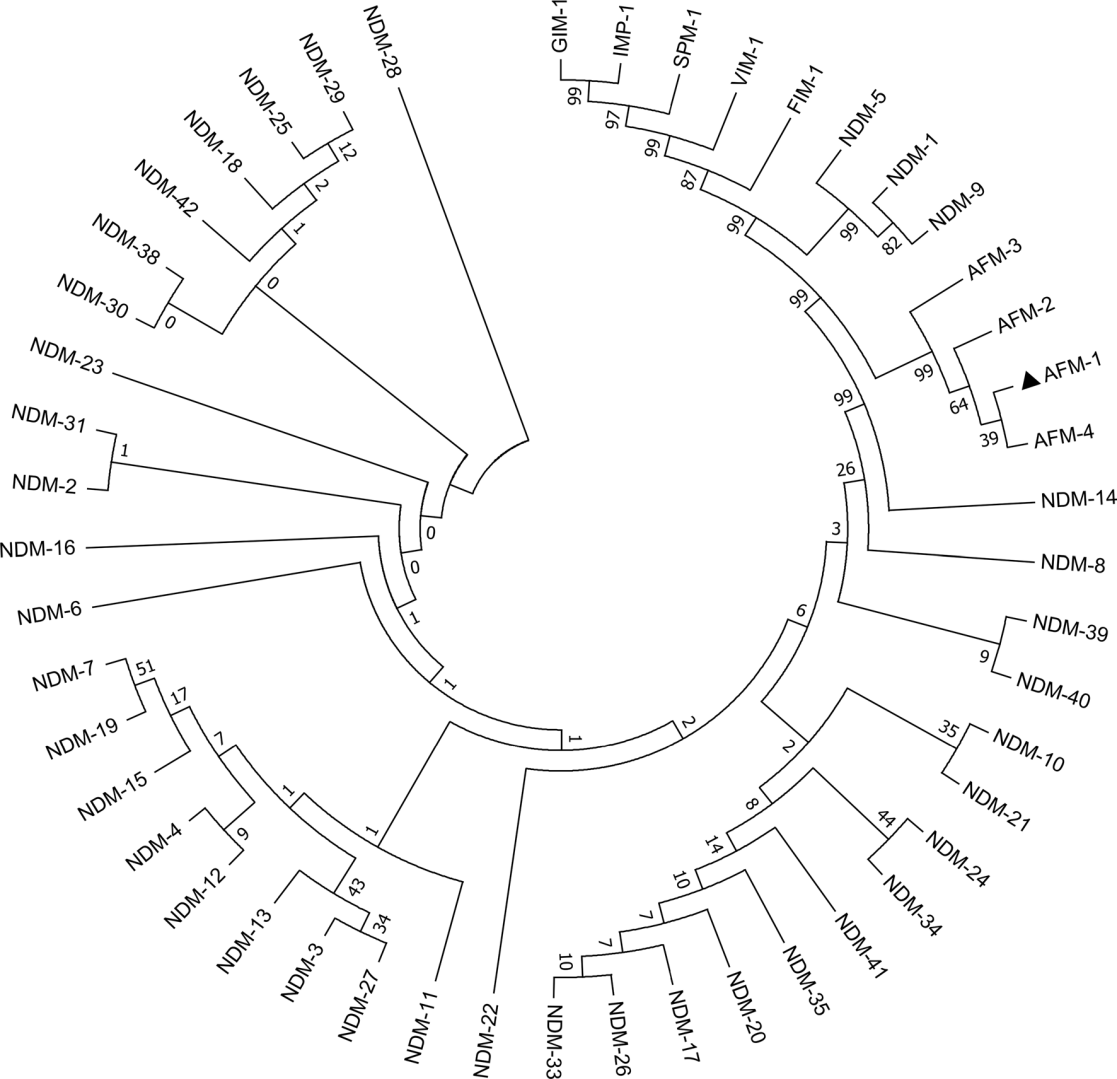
The phylogenetic tree of *bla*_AFM_ gene and common class B1 carbapenemases gene. Note: *bla*_AFM-1_ gene is highlighted by a black triangle symbol.

Before the occurrence variants of *bla*_AFM-1_, by comparison with several B1 carbapenemases genes, *bla*_AFM-1_ showed the highest similarity to *bla*_NDM_, with 93% query coverage and 86% identity at the nucleotide level, followed by *bla*_ANA-1_ and *bla*_SPN79-1_ (both 51% homology with *bla*_AFM-1_ gene at the nucleotide level) (data do not show). Phylogenetic tree analysis (MEGA 7.0) also revealed that the *bla*_AFM-1_ gene was close to *bla*_NDM_ gene family (Fig. 1), which is in line with previous studies [12]. The phylogenetic tree of AFM-1 amino acids and other common B1 metalloenzyme amino acids can be seen in the Additional file 1: Fig. S1. Over time, three subtypes of *bla*_AFM_ genes occurred. Compared with the *bla*_AFM-1_ gene, the *bla*_AFM-2_, *bla*_AFM-3_, and *bla*_AFM-4_ genes have 99% homology with the *bla*_AFM-1_ gene at the nucleotide level, and only a single base mutation (C → T), and the mutation sites are located at the 44th, 37th, 40th position, respectively, and the mutated amino acid corresponding to A14V, P12S, P12L, respectively.

**Fig. 2:**
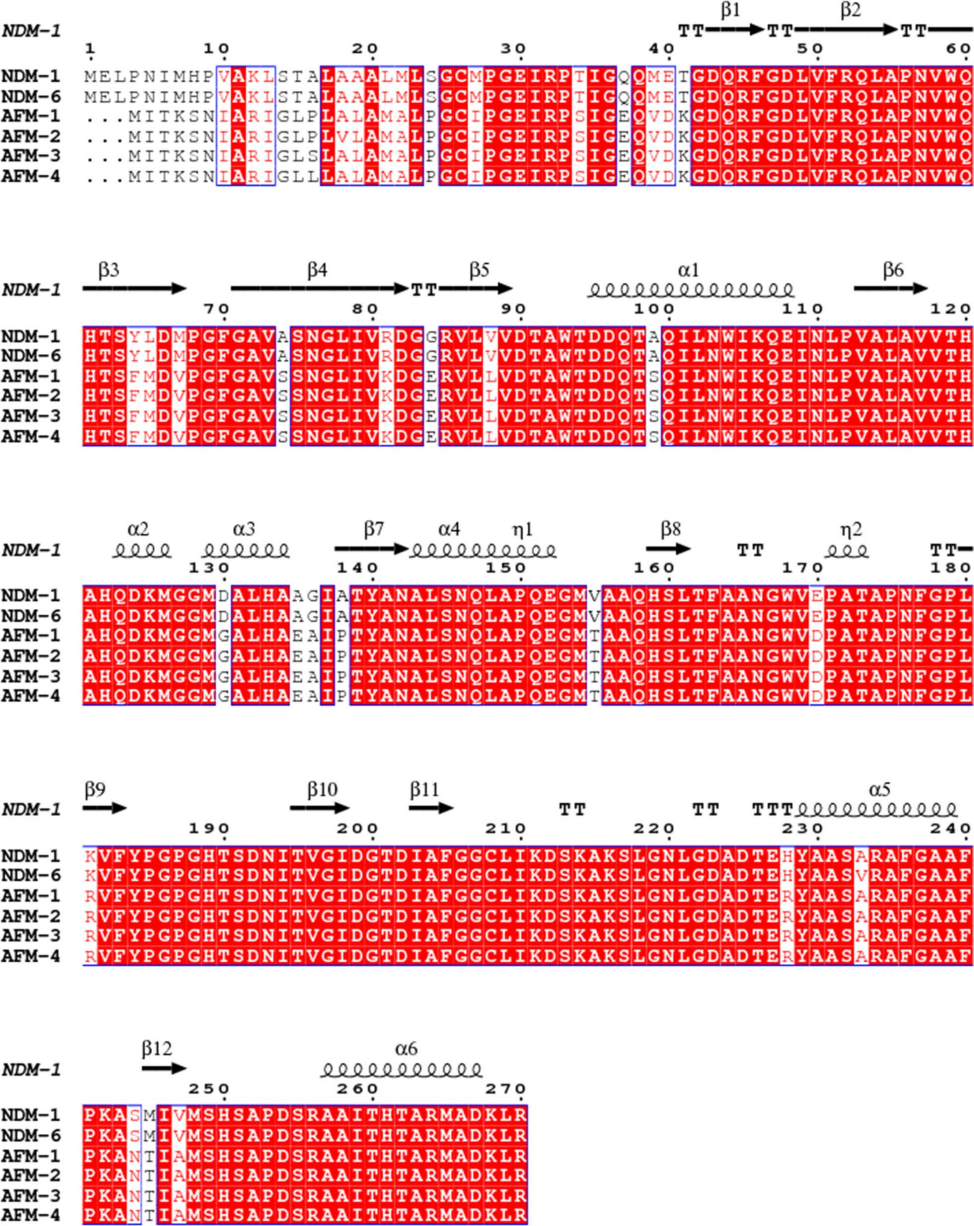
Amino acid alignment among NDM-1 with NDM-6, AFM-1 ~ AFM-4. Note: NDM-1 is at the top of the sequence listed

The *bla*_AFM-1_ gene contained 804 bp and encoded a protein of 267 amino acids with a molecular mass of approximately 28 KDa. Compared with NDM-1 and NDM-6, AFM differs from NDM by 43 amino acids at the amino acid level (Fig. 2).

### Spatial prediction model of AFM enzymes

AFM-1 was composed of an αβ/βα sandwich structure, with two zinc atoms at its active site (see in Additional file 1: Fig. S2), similar to common B carbapenemases. For NDM-1, the active site around the zinc ions was surrounded by amino acids, including His120, His122, His189, His250, Cys208, and Asp124 [23, 24]. While for AFM-1, the key zinc ions coordinating residues were His117, His119, His186, Asp121, Cys205, and His247.

### WGS of *A. faecalis* AN70

**Fig. 3:**
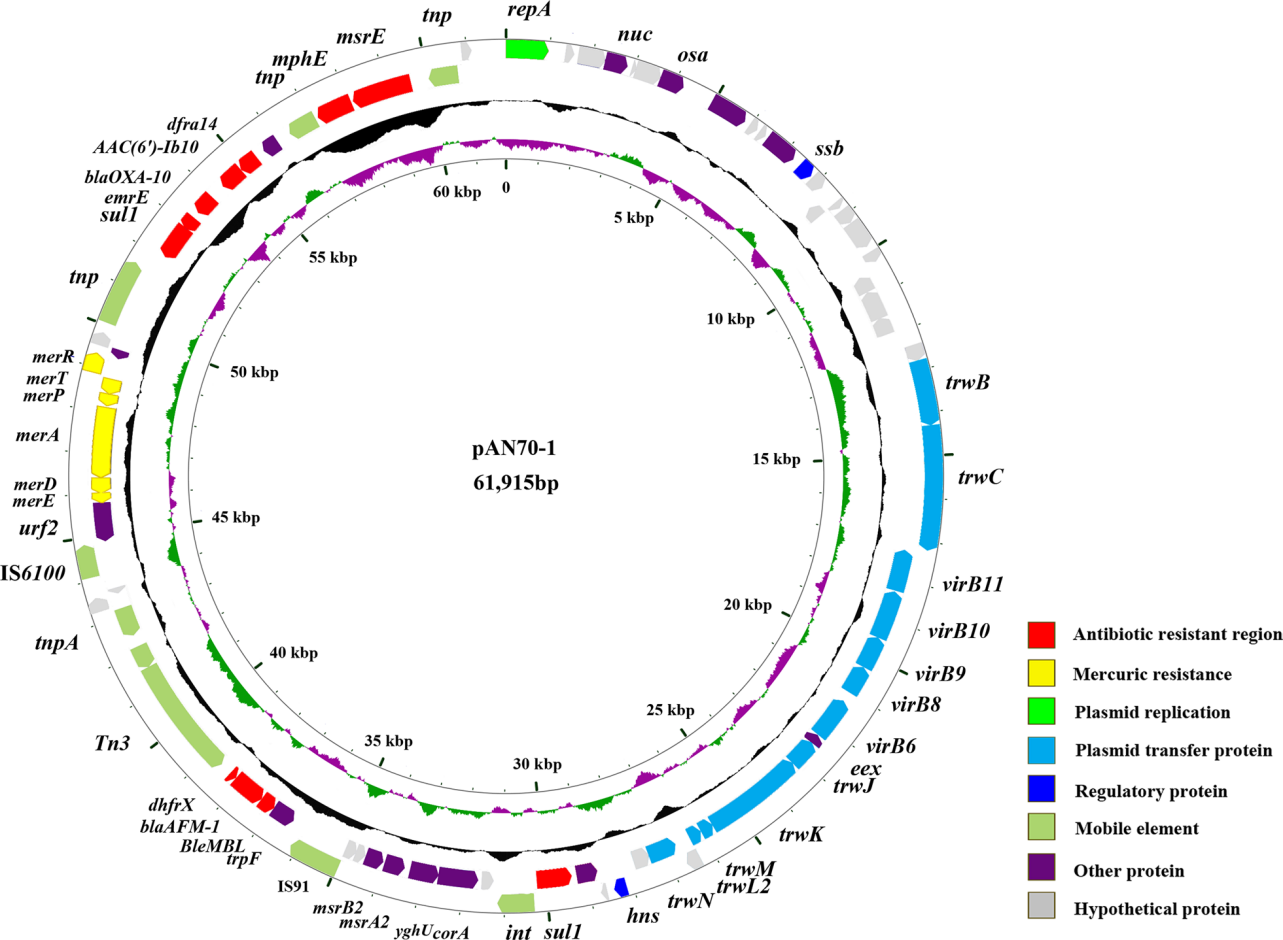
Circle Diagram of pAN70-1

*A. Faecalis* AN70 contained a 3922717 bp chromosome with 50.7% G + C content and a 61,915 bp plasmid (pAN70-1) with 58.1% G + C content. The chromosome of AN70 contained 3,640 coding genes (it also harbored six resistance genes, including *strA*, *strB*, *sul1*, *catB3*, *bla*_OXA-21_, and *AAC (6′)-IIa*), including a new class 1 integron consisting of *aacA3*-*bla*_OXA-21_-*catB3*-*dfrA1b*, termed In1675 (http://integrall.bio.ua.pt/?acc=CP036294), which promoter is “PcWTGN-10 + P2”. pAN70-1 contained 81 coding genes, including 10 ARGs (*bla*_AFM-1_, *msrE*, *mphE*, *dfra14*, *aac(6’)-Ib*, *bla*_OXA-10_, *emrE*, *sul1*, *dhfrX*, *ble*_MBL_) (Fig. 3). Of note, the *bla*_AFM-1_ gene was first identified by us and was submitted to the NCBI database under accession number MK143105.1, and pAN70-1 belongs to the IncW plasmid.

### WGS of *C. testosteroni* NFYY023, *B. trematum *E202, *S. maltophilia* NCTC10498

*C. testosteroni* NFYY023 contained a 4136480 bp chromosome with 59.43% G + C content, and a 89553 bp truncate plasmid (pNFYY023-1) with 57.66% G + C content, a 17,394 bp plasmid (pNFYY023-2) with 52.34% G + C content, and a 6656 bp plasmid (pNFYY023-3) with 52.52% G + C content. ARGs located on *C. testosteroni* NFYY023 chromosome were *cmxA*, *tetG*, *dfrV*, *sul1*, *aadA5*, *aadA3*, *floR*, *aadA1*, *aac(6')-Ib9*, while *bla*_AFM-1_, *ble*_MBL_, *sul1*, *strA*, *strB* etc. ARGs carried on pNFYY023-1.

*B. trematum* E202 contained a 4457823 bp chromosome with 65.47% G + C content. ARGs carried on chromosomes were *aadA2*, *sul1*, *floR*, *ble*_MBL_, *bla*_AFM-1_, *mphA*, *aadA*, *cmlA5*, *AAC(6′)-IIa*.

*S. maltophilia* NCTC10498 contained a 4928653 bp chromosome with 66.25% G + C content. ARGs carried on chromosomes were *bla*_AFM-1_, *ble*_MBL_, *smeR*, *AAC(6')-Iz*, *facT*, *smeF*, *smeE*, *smeD*, *smeC*, *smeB*, *smeA*, *smeS*, etc.

### Cloning expression

**Fig. 4:**
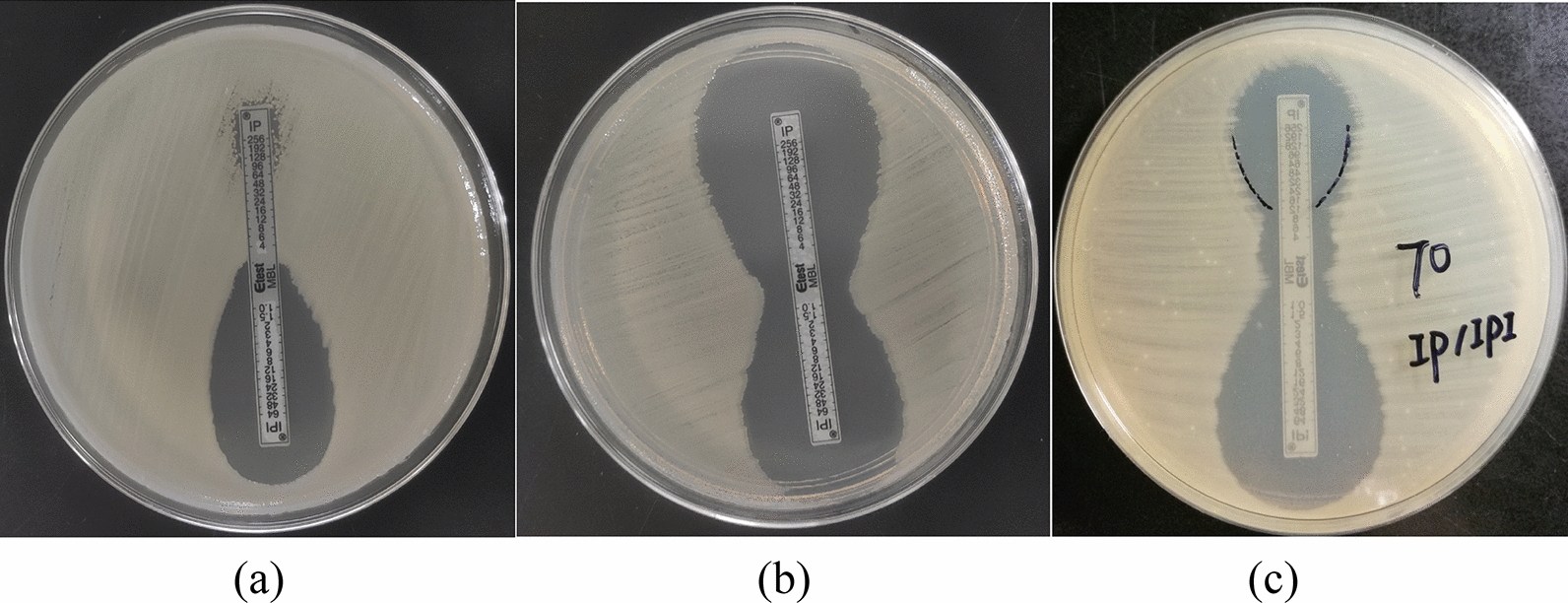
MBL-producing test results. Note: The results of MBL of *A. faecalis* AN70 strain was shown in (**a**), the results of MBL of *E.coli* J53 was indicated in (**b**), the results of MBL of pET-28a( +)-AFM-1-Top10 clone was highlighted in (**c**)

pET-28a( +)-AFM-1-Top10 clone and pET-28a( +)-NDM-1-Top10 clone were successfully constructed, and the PCR/sequencing of these clones using common primers showed that pET-28a( +)-AFM-1-Top10 clone harbored *bla*_AFM-1_ gene, while pET-28a( +)-NDM-1-Top10 clone carried *bla*_NDM-1_ gene. Etest exhibited that the pET-28a( +)-AFM-1-Top10 clone non-susceptible to carbapenems, which demonstrated AFM-1 has the ability to hydrolyze carbapenem agents (Fig. 4).

### Phenotype of AFM-1

Carba NP test showed that AFM-1-producing strains were positive, which was consistent with the positive control (NDM-1-producing strain) (Additional file 1: Table S3), which indicates AFM-1 has the activity of carbapenemase.

### Assessment of horizontal transmission of *bla*_AFM-1_ gene

Transconjugants were grown on MH agar plates containing 4 µg/ml meropenem and 150 µg/ml sodium azide. PCR analysis and sequencing using AFM-1 primers confirmed that the transconjugants contained *bla*_AFM-1_ gene. The pAN70-1 plasmid was successfully transferred from isolate AN70 into *E.coli* J53 by conjugation, at a frequency of 1.3 × 10^−5^. The MIC of meropenem for transconjugant (*E.coli* J53-AN70) was higher than *E.coli* J53 (Table 2). Conjugation assay was carried out on pNFYY023-1 according to the same procedure. Unfortunately, the pNFYY023-1 plasmid failed to complete the conjugation assay.

**Table 2:** MICs (mg/L) of AN70, *E.coli* J53, *E.coli* J53 -AN70

Isolates	IPM	MEM	PIP	CAZ	FEP	CTX	SAM	CPS	TZP	XL	TGC	CST
AN70	8	16	256	256	256	256	256	256	256	256	8	0.75
*E.coli* J53	0.25	0.064	1	0.125	0.064	0.064	8	0.094	1	3	0.25	0.25
*E.coli* J53-AN70	8	8	192	128	16	64	256	256	256	256	0.25	0.25

*IPM* imipenem, *MEM* meropenem, *PIP* piperacillin, *CAZ* ceftazidime, *FEP* cefepime, *CTX* cefotaxime, *SAM* ampicillin/sulbactam, *CPS* cefoperazone/sulbactam, *TZP* piperacillin/tazobactam, *XL* Amoxicillin clavulanic acid, *TGC* Tigecycline, *CST* colistin

### Genetic environment of *bla*_AFM_ gene

**Fig. 5:**
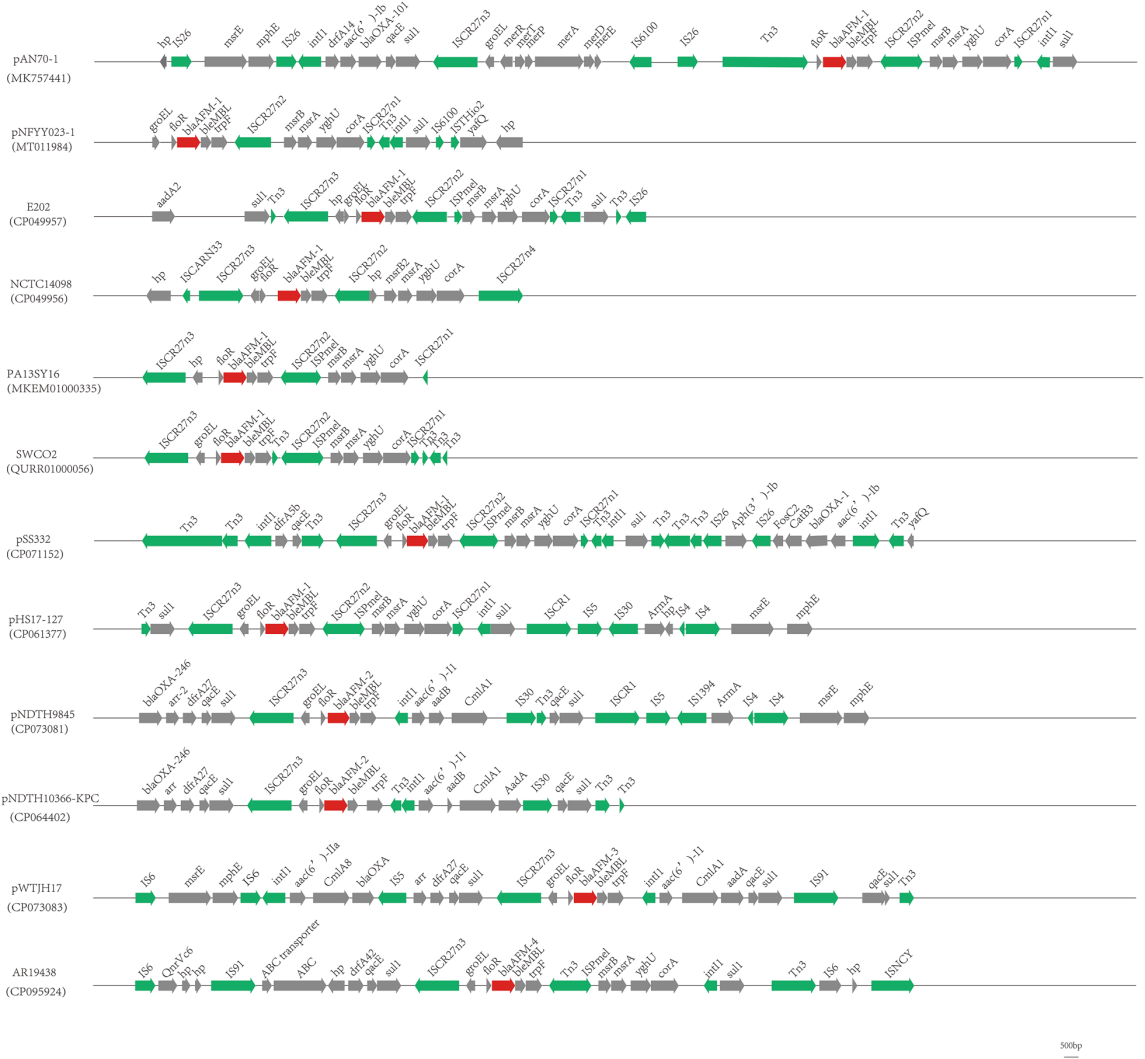
A schematic presentation of the genetic context of *bla*_AFM_ gene. *bla*_AFM_ gene was highlighted in red arrow, other antibiotic resistance genes were indicated by gray arrow, mobile genetic elements were showed by green arrow

By retrieving from the NCBI database, we found several sequences carrying the *bla*_AFM_ genes as follows: the pAN70-1 plasmid of *A. faecalis* AN70 strain (this study), a pNFYY023-1 plasmid from *C. testosteroni* NFYY023 strain (this study), two chromosomes of *B. trematum* E202 strain and *S. maltophilia* NCTC10498 strain (this study), pSS332-218 K plasmid from *A. hydrophila* SS332 strain [11], a pHS17-127 plasmid from carbapenem-resistant *P. aeruginosa* HS17-127 strain [12], PA13SY16 of *P. aeruginosa* strain (Accession NO. MKEM01000335), SWCO2 of *C. testosteroni* strain (Accession NO. QURR01000056)*.* Plasmids pNDTH10366-KPC and pNDTH9845 harbored *bla*_AFM-2_ from the *P. aeruginosa* strain [13]*.* Plasmid pWTJH17 that carried *bla*_AFM-3_ isolated from *P. aeruginosa* strain [13]*.* Plasmid pAR19438 that carried *bla*_AFM-4_ recovered from the *P. aeruginosa* strain [14]*.* Blastn analysis among these sequences and genetic context among *bla*_AFM_ gene were displayed in Fig. 5. All of these sequences contain the component of “*floR*-*bla*_AFM_-*ble*_MBL_-*trpF*”. Insertion sequences analysis revealed that all sequences contained an ISCR27-like module, which was named ISCR27n1, ISCR27n2, ISCR27n3, and ISCR27n4. Compared with ISCR27 sequence, ISCR27n1-4 had 98.67%, 94.42%, 95.33%, 97.17% identity with ISCR27, respectively.

### Comparative genome analysis of plasmid pAN70-1

Plasmid pAN70-1 (Sequence ID: MK089784.1), which plasmid was the first to report to harbor the *bla*_AFM-1_ gene. Homology comparison by Blastn revealed that seven plasmids share > 50% coverage with the pAN70-1 sequence, which was pPROV002-IMP (Sequence ID: MH882484.1), pMAK3 (Sequence ID: AB366442.1), p538_S (Sequence ID: AP025181.1), R388 (Sequence ID: BR000038.1), pMTY10660_IncW (Sequence ID: P018350.1), pHH2-227 (Sequence ID: JN581942.1), and IncW pIE321 (Sequence ID: EF633507.1). Above all these seven sequences, pPROV002-IMP had the highest coverage (59%) with pAN70-1.

The common feature of the pAN70-1 sequence and the pPROV002-IMP sequence is that they both harbored the B1 subclass metalloenzyme gene, that is, the pAN70-1 sequence carries the *bla*_AFM-1_ gene, while the pPROV002-IMP sequence carries the *bla*_NDM-1_ and *bla*_IMP-1_ gene. Meanwhile, these two plasmids have the same replication repA, and they all contain the type IV secretion system complex VirB11, VirB10, VirB9, VirB8, VirB6, VirB4, VirB3, and VirB1 in plasmid conjugal transfer region.

The difference between the pAN70-1 sequence and the pPROV002-IMP sequence is that the pPROV002-IMP sequence without mercury resistance-related genes (*merR*, *merT*, *merP*, *merD*, *merA* and *merE*, etc.), and pPROV002-IMP didn’t form a “*floR*-*bla*_AFM-1_-*ble*_MBL_-*trpF*” composite-like module.

Using pAN70-1 plasmid (harboring *bla*_AFM-1_ gene) as a reference, the homologous identity of pNDTH9845 plasmid (carrying *bla*_AFM-2_ gene), pWTJH17 plasmid (carrying *bla*_AFM-3_ gene) and AR19438 plasmid (carrying *bla*_AFM-4_ gene) were 44.18%, 50.8%, and 53.32%, respectively.

## Discussions

The *Bla*_AFM-1_ gene was first designated to NCBI by us and has been detected in several cities in China. To date, four AFM-type enzymes have been submitted to NCBI. In this study, we described a MBL AFM-1, before the emergence of AFM-2 ~ AFM-4, AFM-1 showed the closest relative to the NDM family, which shares 86% identical with NDM-1, however, they appear to locate at different branches in the phylogenetic tree. Blastn revealed that *bla*_AFM_ possesses 804 bp encoding a protein of 267 amino acids. Carba NP test showed AFM-1 has carbapenemase activity. *E.coli Top10* producing AFM-1 elevated MICs of common beta-lactams, and the Etest strip confirmed the presence of MBL. Spatial structure prediction showed that the active site of AFM-1 enzyme has two zinc ions, which further proves it belongs to subclass B carbapenemase.

Based on the analysis of the enzyme kinetic parameters of Minggui Wang et al. [12] and Yunsong Yu et al. [13], we found that AFM-1, like NDM-1, can hydrolyze three classes of beta-lactams, including penicillins, cephalosporins, and carbapenems. Compared the kinetic parameters of enzyme AFM-1 to substrate imipenem, meropenem, cefepime, and ceftazidime, we found that AFM-1 had the highest affinity for substrate meropenem, further cefepime, ceftazidime, and the lowest affinity for substrate imipenem, which showed by Km value. However, the results of catalytic efficiency (Kcat/Km) about these four substrates are quite different according to Minggui Wang et al. and Yunsong Yu et al. Minggui Wang suggested that the hydrolysis efficiency of the substrate from high to low is ceftazidime, cefepime, imipenem, meropenem, respectively. Yunsong Yu concluded that the hydrolysis efficiency of the substrate from high to low is imipenem, ceftazidime, meropenem, cefepime, respectively. In conclusion, for the AFM-1 enzyme, imipenem has lower affinity but higher catalytic efficiency than meropenem.

AFM carbapenemase was first recovered from *A. faecalis*, which was collected from feces in Guangzhou at first, followed by discovering in *C. testosteroni*, *B. trematum* and *S. maltophilia* strains also originated from feces sample in Guangzhou. Over time, it was recovered from *A. hydrophila* isolated from a fecal sample in Lishui [11], *P. aeruginosa* isolated from a urine sample in Shanghai [12], *P. aeruginosa* obtained from a venous blood sample in Hangzhou [13]. So far, all the species carrying the target gene are located in China, but the distribution of the *bla*_AFM_ gene in different species further implies the potential risk of the introduction of the *bla*_AFM_ gene into clinically important strains and the dissemination to other countries. We underline the diversity and variability of the hosts of the *bla*_AFM_ gene.

The above strains all went through WGS, and blastn analysis revealed that the sequence surrounding *bla*_AFM-1_ was “*floR*-*bla*_AFM_-ble_MBL_-*trpF*”. At the nucleotide level, the genetic environment of *bla*_AFM-1_ was 99% identical to that of *rettgeri Providencia* (MH882484.1) isolated in Lanzhou, China, which did not harbor the *bla*_AFM-1_ gene. The analysis of the AFM enzyme gene genetic environment showed that almost all sequences carried an ISCR27-like element, which might be related to its acquisition mobilization of the *bla*_AFM_ gene to spread to other strains, which like the mobilization of the *bla*_NDM-1_ gene was also related to ISCR27 element [25, 26].

AFM-1 are of plasmid and chromosomal origin. Expression of AFM-1 in top10 cells conferred resistance to carbapenems. What’s worrisome, conjugation assay successfully mediated by plasmid among *bla*_AFM-1_ ~ *bla*_AFM-4_ suggested that *bla*_AFM_ genes are readily to transfer via plasmid [12–14]. Therefore, AFM-type MBLs have evolved and AFM-producing strains needs to be concerned in China, even in the global future.

We are currently conducting molecular epidemiological studies of Gram-negative strains in China to explore the prevalence of AFM genes in clinical and non-clinical isolates, continuous surveillance has very significance for the prevention and control of the AFM gene dissemination.

## Conclusions

*Bla*_AFM-1_ gene was detected in several different species in Guangzhou, China. The AFM-1 enzyme encoded by *bla*_AFM-1_ gene can hydrolyze carbapenem antibiotics and can be transferred horizontally through plasmid conjugation, thereby conferring resistance to carbapenem-susceptible strains.

## Supplementary Information

**Additional file 1: Table S1.** AST of clinical isolates of Klebsiella pneumoniae 10003730. **Table S2.** AST of clinical isolates of Escherichia coli 10004114. **Table S3.** The results of Carba NP test. **Fig S1.** The phylogenetic tree of amino acids between AFM and common class B1 carbapenemases **Fig S2.** Three-dimensional structure of AFM and NDM carbapenemases.

## Data Availability

This article contains all the research data and materials of this research.

## Abbreviations

MBLs: Metallo-β-lactamases
IMP: Imipenemase metallo-β-lactamase
VIM: Verona integron-encoded metallo-β-lactamase
NDM: New Delhi metallo-β-lactamase
MGEs: Mobile genetic elements
IS: Insertion sequences
ARGs: Antimicrobial resistance genes
WGS: Whole genome sequencing
AST: Antimicrobial susceptibility testing
*A*. *Faecalis*: *Alcaligenes* *faecalis*
*B*. *Trematum*: *Bordetella* *trematum*
*C*. *Testosteroni*: *Comamonas* *testosteroni*
*S*. *maltophilia*: *Stenotrophomonas* *maltophilia*
*A*. *Hydrophila*: *Aeromonas* *hydrophila*
*P*. *Aeruginosa*: *Pseudomonas* *aeruginosa*
