# Supplementary material for: Characterization of blaAFM-1-positive carbapenem-resistant strains isolated in Guangzhou, China
Source: Ann Clin Microbiol Antimicrob. 2023 May 17;22:40. doi: 10.1186/s12941-023-00592-0 (PMC10189940; doi:10.1186/s12941-023-00592-0)
Supplement: Supplementary file 1 — Additional file 1: Table S1. AST of clinical isolates of Klebsiella pneumoniae 10003730. Table S2. AST of clinical isolates of Escherichia coli 10004114. Table S3. The results of Carba NP test. Fig S1. The phylogenetic tree of amino acids between AFM and common class B1 carbapenemases Fig S2. Three-dimensional structure of AFM and NDM carbapenemases. [file 12941_2023_592_MOESM1_ESM.doc]

**Additional file materials**

Table S1 AST of clinical isolates of *Klebsiella pneumoniae* 10003730.

| Antibiotics | MICs (μg/ml) | breakpoint | interpretation |
| --- | --- | --- | --- |
| Gentamin | <=2 | <=4 | S |
| Amikacin | <=8 | <=16 | S |
| Piperacillin | <=4 | <=8 | S |
| Ampicillin/Sulbactam | <=4/2 | <=8/4 | S |
| Piperacillin/Tazobactactam | <=4/4 | <=8/4 | S |
| Amoxicillin/Clavulanic | <=4/2 | <=21 | S |
| Cefoperazone/Sulbactam | <=2 | <=15 | S |
| Cefazolin | <=1 | <=2 | S |
| Cefepime | <=1 | <=2 | S |
| Cefotaxime | <=1 | <=1 | S |
| Ceftazidime | <=1 | <=4 | S |
| Imipenem | <=1 | <=1 | S |
| Meropenem | <=1 | <=1 | S |
| Ciprofloxacin | <=0.25 | <=0.25 | S |
| Levofloxacin | <=0.5 | <=0.5 | S |
| Moxifloxacin | <=1 |  | S |
| Trimethoprim/Sulfamethoxazole | <=0.5/9.5 | <=2/38 | S |
| Tetracycline | <=4 | <=4 | S |
| Tigecycline | <=2 | <=2 | S |
| Aztreonam | <=2 | <=4 | S |
| Chloramphenicol | <=4 | <=8 | S |
| colistin | <=0.5 | <=2 | S |

Table S2 AST of clinical isolates of *Escherichia coli* 10004114.

| Antibiotics | MICs (μg/ml) | breakpoint | interpretation |
| --- | --- | --- | --- |
| Gentamin | <=2 | <=4 | S |
| Amikacin | <=8 | <=16 | S |
| Ampicillin | <=4 | <=8 | S |
| Piperacillin | <=4 | <=8 | S |
| Ampicillin/Sulbactam | <=4/2 | <=8/4 | S |
| Piperacillin/Tazobactactam | <=4/4 | <=8/4 | S |
| Amoxicillin/Clavulanic | <=4/2 |  | S |
| Cefoperazone/Sulbactam | <=2 | <=15 | S |
| Cefepime | <=2 | <=2 | S |
| Cefotaxime | <=1 | <=1 | S |
| Ceftazidime | <=1 | <=4 | S |
| Imipenem | <=1 | <=1 | S |
| Meropenem | <=1 | <=1 | S |
| Ciprofloxacin | <=0.25 | <=0.25 | S |
| Levofloxacin | <=0.5 | <=0.5 | S |
| Moxifloxacin | <=1 |  | S |
| Trimethoprim/Sulfamethoxazole | <=0.5/9.5 | <=2/38 | S |
| Tetracycline | <=2 | <=4 | S |
| Aztreonam | <=2 | <=4 | S |
| Chloramphenicol | <=4 | <=8 | S |
| colistin | <=0.5 | <=2 | S |

Table S3 The results of Carba NP test.

| Isolates | strain | gene | results |
| --- | --- | --- | --- |
| AN70 | *Alcaligenes faecalis* | *bla*_AFM-1_ | + |
| 3 | *Escherichia coli* | *bla*_NDM-1_ | + |
| NFYY023 | *Comamonas testosteroni* | *bla*_AFM-1_ | + |
| E202 | *Bordetella trematum* | *bla*_AFM-1_ | + |
| NCTC10498 | *Stenotrophomonas maltophilia* | *bla*_AFM-1_ | + |
| 10003730 | *Klebsiella pneumoniae* | */* | - |
| 10004114 | *Escherichia coli* | */* | - |

Fig. S1 The phylogenetic tree of amino acids between AFM and common class B1 carbapenemases


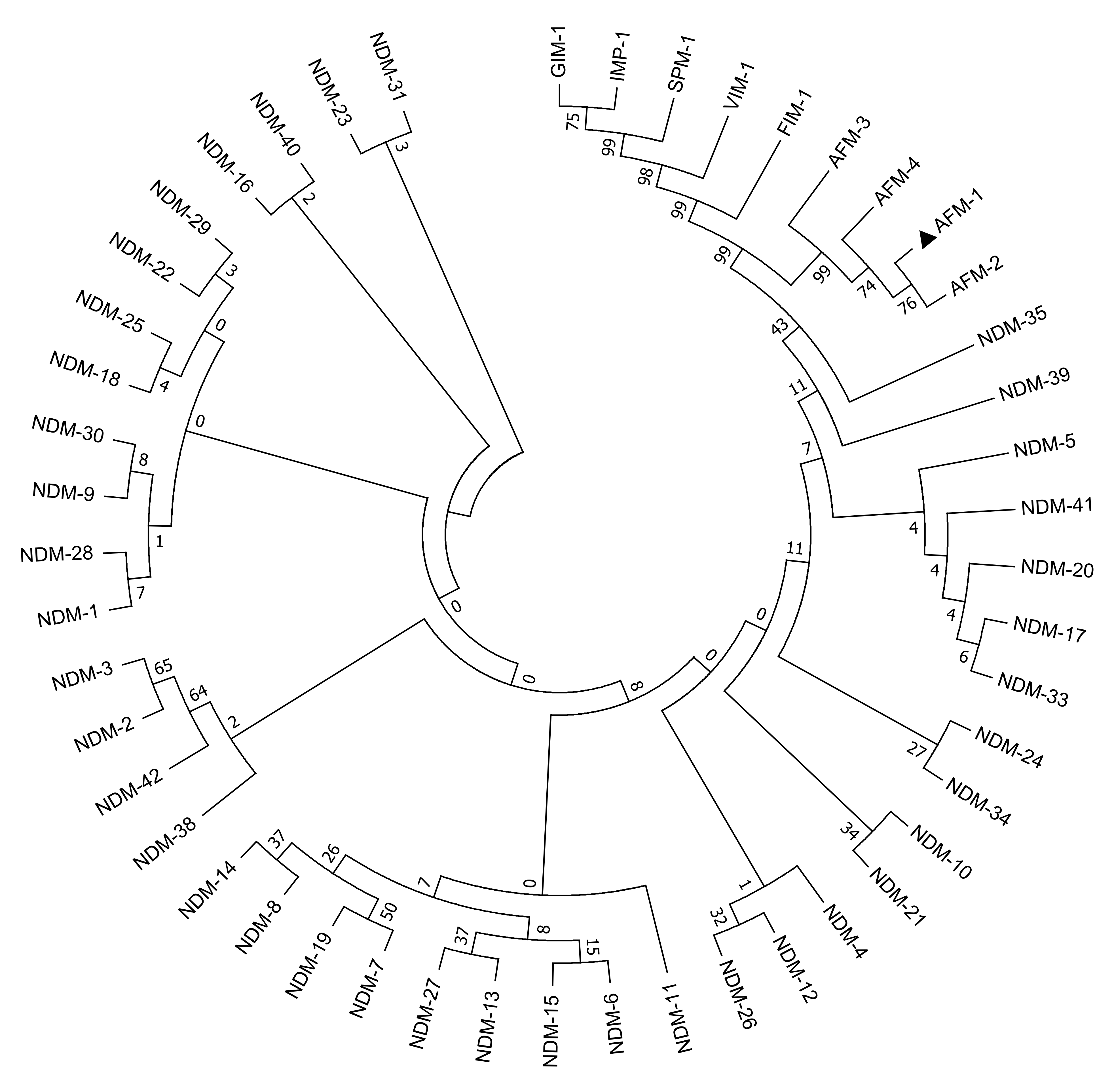


Note: AFM-1 enzyme is highlighted by a black triangle symbol.

Fig. S2 Three-dimensional structure of AFM (1-4) and NDM (1/6) carbapenemases.


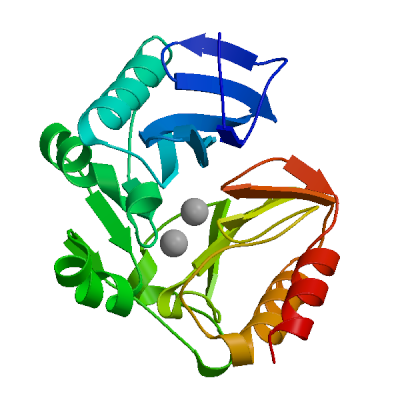

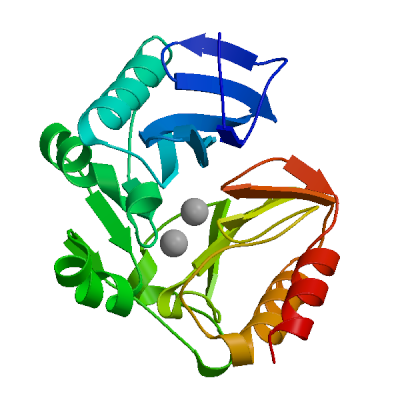


AFM-1 AFM-2


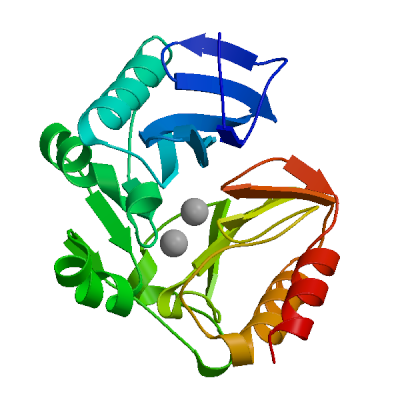

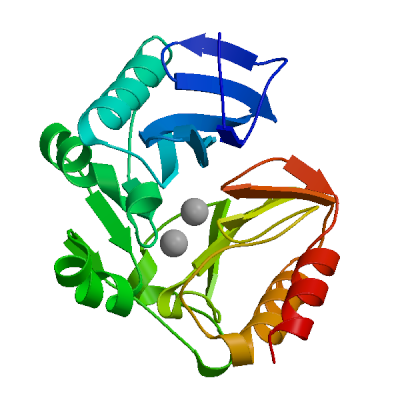


AFM-3 AFM-4


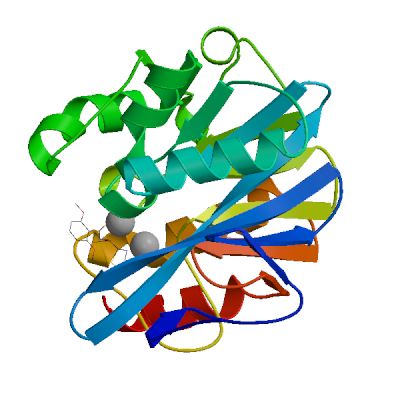

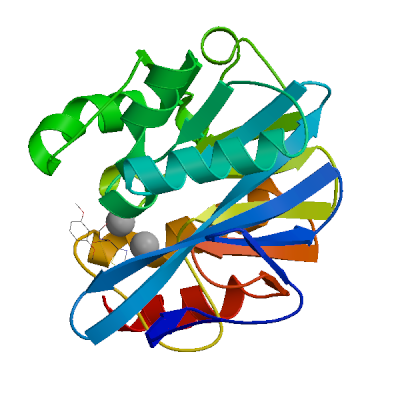


NDM-1 NDM-6

Note: Gray spheres represent two zinc ions.
